# Early Childhood Anxiety and Maternal Factors: Associations with State and Trait Anxiety in a Greek Cohort of Preschoolers

**DOI:** 10.3390/medsci14010092

**Published:** 2026-02-15

**Authors:** Exakousti-Petroula Angelakou, Sousana K. Papadopoulou, Eleni Pavlidou, Aikaterini Louka, Konstantina Gerothanasi, Constantinos Giaginis

**Affiliations:** 1 Department of Food Science and Nutrition, School of Environment, University of Aegean, 81400 Lemnos, Greece; pangelakou@aegean.gr (E.-P.A.); elen.p.pavl@gmail.com (E.P.); loukathy612@gmail.com (A.L.); 2 Department of Nutritional Sciences and Dietetics, School of Health Sciences, International Hellenic University, 57400 Thessaloniki, Greece; souzpapa@gmail.com (S.K.P.); kgero@nutr.teithe.gr (K.G.)

**Keywords:** preschool children, child anxiety, state anxiety, trait anxiety, maternal factors, early childhood mental health

## Abstract

Background/Objective: Anxiety symptoms in preschool children represent early indicators of potential mental health vulnerabilities. Maternal psychological, sociodemographic, lifestyle and dietary factors may be associated with child emotional development; however, evidence regarding their independent contributions to distinct dimensions of child anxiety (trait vs. state) remains limited. This study aimed to examine maternal factors associated with preschool children’s trait and state anxiety. Methods: In this cross-sectional study conducted in Greece, 200 preschool-aged children and their mothers were assessed. Maternal demographic, socioeconomic, anthropometric, lifestyle, dietary, and psychosocial characteristics were evaluated using validated instruments, including the Mediterranean Diet Score (MedDietScore), Beck Depression Inventory–II (BDI-II), and the State–Trait Anxiety Inventory short form (STAI-6). Children’s trait and state anxiety were assessed using the State–Trait Anxiety Inventory for Children (STAI-CH). Bivariate analyses were conducted, followed by separate multivariable linear regression models for trait and state anxiety, with covariate selection guided by a directed acyclic graph (DAG). Results: Maternal anxiety was positively associated with children’s state anxiety (B = 1.508, SE = 0.566, β = 0.196, t = 2.666, *p* = 0.008; 95% CI [0.43, 2.66]). Higher maternal educational attainment demonstrated a weak positive association with child state anxiety (B = 1.061, SE = 0.509, β = 0.145, t = 2.086, *p* = 0.038; 95% CI [0.08, 2.09]), which may reflect greater awareness or reporting of child symptoms by more-educated mothers or other unmeasured factors. For trait anxiety, maternal depressive symptomatology exhibited the strongest association (B = 3.578, SE = 0.918, β = 0.276, t = 3.897, *p* < 0.001; 95% CI [1.77, 5.39]), while maternal anxiety was also independently associated with higher trait anxiety (B = 2.088, SE = 0.744, β = 0.194, t = 2.807, *p* = 0.006; 95% CI [0.62, 3.56]). The models explained a modest proportion of variance (R^2^ < 0.15), indicating that most variation in child anxiety does not seem to be fully explained by the specific measured maternal factors. Conclusions: Maternal psychological distress was modestly associated with preschool children’s state and trait anxiety, exhibiting differential patterns across anxiety dimensions. These findings should be interpreted as correlational, with unmeasured contributors such as paternal mental health, family functioning, genetics, and school/peer influences likely playing important roles. Early screening and interventions addressing maternal mental health may support children’s emotional well-being, but further multi-informant and longitudinal research is needed to clarify temporal and causal pathways.

## 1. Introduction

Preschool age represents a critical developmental period for the consolidation of mental health, marked by accelerated neurobiological maturation and heightened neural plasticity within circuits governing emotional regulation and executive functioning [1]. During this stage, children’s psychological well-being emerges through dynamic and reciprocal interactions between the child and multilayered environmental systems, including the family, primary caregivers, and broader sociocultural contexts. From a systemic-developmental perspective, early psychological development reflects the continuous interplay among biological, psychological, and social processes rather than linear, unidirectional pathways [2,3].

Anxiety-related manifestations, including separation anxiety and specific phobias, commonly arise during the preschool years. Clinical anxiety disorders occur in approximately 0.3–7% of children, whereas symptoms are observed in 10–20% [4,5,6,7]. Although mild separation anxiety is developmentally expected, persistence beyond age-appropriate limits or impairment in daily functional indicates clinical relevance [8]. Somatic expressions of anxiety, such as abdominal pain, headaches, and generalized discomfort, are also frequent in preschoolers, reflecting immature fronto-limbic circuitry and limited verbal emotional expression capacities [9,10,11].

Self-regulation constitutes a foundational mechanism supporting emotional stability and psychological resilience in early childhood. These skills emerge through co-regulatory caregiver–child interactions that scaffold emotion modulation and behavioral control [12,13]. Exposure to excessive stress or maladaptive relational environment can disrupt the maturation of neural systems, including the prefrontal cortex and amygdala, resulting in reduced cognitive flexibility, impaired emotion regulation, and increased vulnerability to anxiety [14]. Furthermore, parental psychological distress, inconsistent caregiving patterns, and dysfunctional emotion management strategies have been associated with child impulsivity, emotional dysregulation, and poorer social adaptation [15,16,17].

Differentiating between trait and state anxiety in early childhood is essential Trait anxiety denotes a stable predisposition toward emotional sensitivity and stress reactivity, whereas state anxiety reflects transient responses to immediate stressors [18]. These dimensions may be differentially shaped by maternal, psychological, familial, and socio-economic factors, underscoring the need for multidimensional approaches to understanding child anxiety [19,20,21].

Maternal mental health is a central determinant of children’s psychological outcomes. Maternal postpartum depression and anxiety have been linked to reduced emotional responsiveness, lower-quality mother-child interaction, and diminished emotional support, increasing the likelihood of child irritability, heightened worry, and self-regulation difficulties [22,23,24,25,26,27]. Other maternal psychopathological conditions, including personality disorders and generalized anxiety disorder, are associated with maladaptive caregiving and elevated risk for child internalizing and externalizing difficulties [28,29]. Socio-demographic and economic factors, such as maternal education, family composition, and household income, may further mediate or moderate these associations, while maternal behaviors influencing sleep routines, diet, and daily structure may act as protective or risk factors for anxiety symptoms [17,30,31,32].

The post-COVID-19 period has intensified these concerns, as increases in maternal anxiety and depressive symptoms have been associated with poorer emotional and behavioral outcomes in children [33]. This context highlights the need for contemporary research examining the impact of maternal psychosocial and lifestyle factors on both trait and state anxiety in preschool-aged children.

This study investigates the intergenerational relationships between maternal sociodemographic, lifestyle, and psychosocial characteristics and levels of trait and state anxiety in preschool-aged children within a Greek population. Specifically, it examines: (i) the influence of maternal characteristics on children’s psychological well-being; (ii) the direct associations between maternal factors and the two dimensions of child anxiety; and (iii) the combined contribution of maternal, behavioral, and socio-economic variables to child anxiety outcomes. Through this approach, the study offers a comprehensive and multidimensional understanding of how maternal influences shape early psychological development in children.

## 2. Methods

### 2.1. Study Population

The present investigation employed a cross-sectional descriptive-analytical design to examine maternal and child health characteristics within mother-preschool child dyads. The design enabled the concurrent assessment of psychosocial, behavioral, and nutritional variables within a population subgroup potentially vulnerable to health inequalities, while maintaining feasibility in terms of participant retention and field logistics [34,35]. However, the cross-sectional structure precludes temporal ordering, limiting casual inference and leaving the possibility of residual confounding [36].

The study sample comprised 200 preschool-aged children (4.0–6.5 years) and their mothers, who served as primary respondents for questionnaire-based data collection. Recruitment was conducted using a multistage probabilistic sampling framework across public and private early childhood education settings in 13 administrative regions of Greece, capturing substantial geographic, socioeconomic, and cultural heterogeneity. To account for the hierarchical sampling structure, potential clustering at preschools and regional levels was evaluated using intraclass correlation coefficients (ICCs) for both state and trait anxiety outcomes. Observed ICCs were consistently below 0.05, indicating minimal within-cluster dependence and supporting the use of standard linear regression models without multilevel or cluster-robust adjustments.

Data collection was performed over an 18-month period (October 2023–April 2025), through standardized, in-person maternal interviews conducted by trained research personnel. Anthropometric measurements for mothers and children were obtained using calibrated equipment and standardized protocols to ensure inter-observer reliability and measurement accuracy.

Eligibility criteria required participating mothers to have a youngest child aged 4–6.5 years at the time of assessment. Although an a priori sample size calculation was not performed, post-recruitment evaluation of model stability was undertaken. The number of covariates included in multivariable models was restricted to maintain an appropriate observation-to-parameter ratio, thereby minimizing overfitting risk. Parameter estimate precision was additionally evaluated through confidence interval width. While some estimates demonstrated moderate variability, overall model outputs remained statistically stable and interpretable. These limitations are acknowledged, and larger-scale studies are recommended to enhance robustness and generalizability of findings.

All procedures adhered to established ethical research standards. Participation was voluntary, and written informed consent was obtained from all mothers prior to enrollment. Participants received individualized feedback regarding mental health and lifestyle factors to promote engagement and evidence-informed health awareness [37]. Data management procedures complied fully with the General Data Protection Regulation, ensuring secure storage, controlled access, and anonymization of identifiable information [38]. Ethical approval was granted by the Ethics Committee of the University of the Aegean (Approval No. 47/21.9.2023). All procedures were conducted in accordance with the principles of the Declaration of Helsinki [39].

### 2.2. Study Design

**Figure 1:**
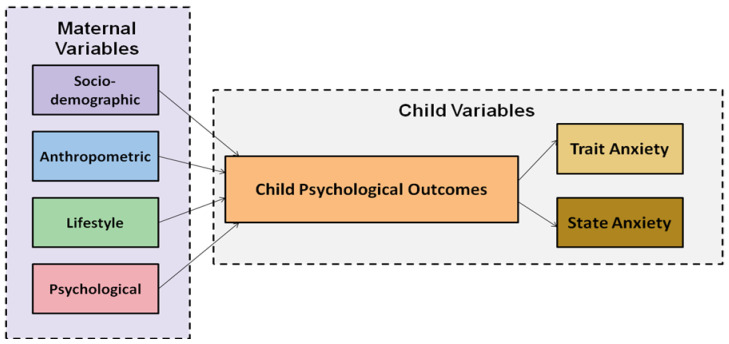
Conceptual framework of maternal influences on preschool children’s trait and state anxiety.

The present study utilized a quantitative, non-experimental, descriptive-correlational design, to examine multidimensional associations between maternal biological, behavioral, and psychosocial characteristics and preschool children’s psychological health (Figure 1). This approach enabled the concurrent evaluation of multiple exposure domains within a population subgroup potentially susceptible to health inequalities, while maintaining methodological feasibility and minimizing participant burden compared with longitudinal follow-up designs [34,35]. However, due to the lack of temporal sequencing, causal inference is limited, and residual confounding cannot be fully excluded [36].

Primary data collection was conducted through standardized maternal interviews performed in familiar residential environments to facilitate participant comfort and optimize response accuracy [40]. Interviews were administered by trained research personnel using structures data collection protocols to enhance reproducibility and reduce systematic reporting bias. Recorded variables included maternal demographic characteristics, household socioeconomic parameters, and core child characteristics, including age and biological sex, alongside parental educational attainment, employment status, and perceived economic adequacy.

Anthropometric measurements were obtained using internationally standardized procedures and regularly calibrated equipment. Maternal body weight, height, and central adiposity indices were recorded and subsequently used to calculate Body Mass Index (BMI) and Waist-to-Hip Ratio (WHR) values. Self-reported maternal weight prior to pregnancy and immediately before delivery was additionally collected to estimate pre-pregnancy BMI, pre-delivery BMI, and Gestational Weight Gain (GWG). Maternal BMI categorization followed World Health Organization (WHO) reference criteria. To improve measurement precision, each anthropometric parameter was recorded in duplicate and averaged prior to analysis.

Maternal health-related exposures included dietary quality measures, behavioral lifestyle characteristics, and mental health symptom measures. These comprised adherence to the Mediterranean dietary pattern, physical activity participation, smoking habits, presence of chronic medical conditions, pharmacological treatment, depressive symptom burden, and anxiety symptom severity. Child-level variables included physician-diagnosed medical conditions, neurodevelopmental or behavioral diagnoses, and anxiety symptom expression.

Psychological and dietary constructs were quantified using four (4) psychometrically validated instruments previously standardized for use within Greek populations: the MedDietScore, Beck Depression Inventory-II (BDI-II), Six-Item State-Trai Anxiety Inventory (STAI-6), and State–Trait Anxiety Inventory for Children (STAI-CH). All instruments demonstrate established construct validity, internal consistency, and diagnostic sensitivity in epidemiological and clinical research contexts. Administration followed a standardized sequence under researcher supervision to support comprehension while minimizing interviewer-induced response bias. No generative AI tools were used during study conception, data acquisition, statistical analysis, or manuscript preparation.

This integrated methodological framework supports comprehensive evaluation of multifactorial maternal influences on early childhood psychological outcomes within a geographically diverse Greek preschool population.

### 2.3. Assessment of Adherence to the Mediterranean Dietary Pattern

Maternal compliance with the Mediterranean Diet (MD) was quantified using the MedDietScore, a previously validated semi-quantitative dietary assessment tool developed to estimate habitual consumption patterns across core components of the MD [41]. The instrument captures intake frequency across eleven (11) major dietary groups, including plant-based foods (fruits, vegetables, legumes, and cereals), fish, and olive oil, as well as animal-derived products such as red and processed meat, poultry, and dairy products, in addition to alcohol consumption.

Responses are converted into component-specific scores reflecting concordance with Mediterranean dietary recommendations, which are subsequently summed to produce a composite dietary quality index ranging from 0–55. Higher total scores indicate stronger adherence to the Mediterranean dietary model

Prior validation studies have supported the construct validity of the MedDietScore, demonstrating significant associations with cardiometabolic risk factors and systemic inflammatory indices [41,42,43,44]. In the current cohort, internal consistency was deemed satisfactory (Cronbach’s α = 0.760), supporting the reliability of this measure for maternal dietary assessment.

### 2.4. Assessment of Depressive Symptoms

Maternal depressive symptoms burden was measured using the Beck Depression Inventory–II (BDI-II), a widely applied standardized questionnaire consisting of 21 items designed to quantify depressive symptom expression during the preceding two weeks [45]. The measure encompasses multiple facets of depression, including affective disturbances, maladaptive cognitive appraisals, and physiological or behavioral symptom presentations. Participants indicate symptom intensity using an ordinal four-level response format (0–3), generating cumulative scores between 0 and 63, where higher totals correspond to greater depressive symptom severity.

The Greek adaptation of the BDI-II exhibits strong psychometric adequacy, including excellent internal consistency (Cronbach’s α = 0.93) and satisfactory validity across clinical and non-clinical populations [46]. Findings from international validation research similarly support the scale’s reliability and diagnostic performance, with internal consistency coefficients generally reported around α ≈ 0.90 across heterogeneous study samples [47]. Within the present study population, the BDI-II demonstrated satisfactory internal consistency (Cronbach’s α = 0.826), supporting its suitability for quantifying depressive symptoms among Greek mothers of preschool-aged children.

### 2.5. Assessment of Anxiety Disorder

Maternal anxiety levels were evaluated using the six-item short-version of the State-Trait Anxiety Inventory (STAI-6), a condensed form of the original 20-item instrument developed to provide rapid yet reliable assessment of anxiety-related emotional states [48]. The scale captures core affective responses associated with anxiety, including feelings of calmness, relaxation, tension, distress, worry, and positive affect. Participants rate each item according to their current emotional experience.

To ensure comparability with the full STAI, raw scores were converted to the conventional STAI metric (range: 20–80), allowing interpretation using established clinical reference thresholds. Based on previously validated criteria, scores within the 34–37 range are considered indicative of typical anxiety levels, whereas values of 38 or higher suggest elevated anxiety symptomatology [49,50].

Previous validation studies have demonstrated satisfactory reliability of the STAI-6, with internal consistency estimates around α = 0.82 and strong agreement with the full-length STAI (r = 0.95) [49]. In the current sample, the scale demonstrated acceptable internal consistency (Cronbach’s α = 0.764), supporting its use for assessing maternal anxiety in this population.

Preschool-aged children’s anxiety levels was evaluated using the State–Trait Anxiety Inventory for Children (STAI-CH), a psychometrically established 40-item assessment tool consisting of two theoretically distinct dimensions: State Anxiety (A-State), representing situationally induced and time-specific anxiety responses, and Trait Anxiety (A-Trait), reflecting relatively enduring predispositional anxiety characteristics [18]. Each item is scored using a three-point Likert response format, producing subscale scores between 20 and 80, while the cumulative anxiety index ranges from 40 to 160. Higher values correspond to elevated anxiety symptomatology.

The Greek adaptation of the STAI-CH has previously demonstrated robust psychometric adequacy, including strong indices of internal consistency and construct validity across validation samples [51]. Considering the developmental characteristics of preschool children, particularly limitations in abstract reasoning, verbal expression, and introspective self-evaluation, the instrument was administered through maternal proxy-report in the present study.

For the A-State dimension, mothers were asked to evaluate their child’s emotional condition at the specific time of the data collection, focusing on immediate affective expressions such as calmness, tension, distress, nervousness, fearfulness, positive mood, worry, or irritability. These items were designed to capture short-term emotional reactivity and context-dependent affective fluctuations. In contrast, A-Trait items required mothers to report the typical frequency with which their child exhibits anxiety-related cognitive, emotional, or physiological patterns, including indecisiveness, persistent worries (e.g., school-related or family-related concerns), heightened sensitivity to social evaluation, or somatic correlates of anxiety such as palmar sweating.

To preserve measurement integrity, no modifications were made to the original item content or response structure. However, standardized explanatory instructions were provided to ensure maternal understanding of the temporal and conceptual differentiation between state-related and trait-related anxiety constructs.

This methodological approach is well supported in the international literature as a valid and reliable means of assessing young children’s emotional and internalizing symptoms when self-report is not developmentally feasible [52,53,54].

Parental proxy-reports have been shown to provide meaningful and reliable representations of children’s anxiety-related behaviors and emotional states, particularly when data collection is conducted under structured and supervised conditions [54]. In the current sample, the STAI-CH demonstrated high internal consistency (Cronbach’s α = 0.858 for the total scale), confirming the reliability of the instrument within a proxy-report framework in a preschool population.

All maternal and child-related assessments were administered by trained personnel following standardized procedures. Completion of the STAI-CH was carried out under the systematic guidance of the principal researcher, a certified early childhood educator with extensive professional experience, who provided clarification solely to facilitate comprehension of questionnaire items without influencing response content. This administration protocol adhered to internationally accepted methodological standards for psychological research involving young children, thereby ensuring the validity and reliability of the collected data [54,55].

### 2.6. Statistical Analysis

Statistical analyses were conducted to characterize the study population and examine associations between maternal characteristics and preschool children’s anxiety outcomes. Continuous variables were assessed for normality using the Kolmogorov–Smirnov test and are reported as mean ± standard deviation (SD) for approximately normally distributed measures or median and interquartile range (IQR) for skewed distributions. Categorical variables are summarized as counts and percentages. Between-group comparisons were performed using Student’s *t*-test for normally distributed continuous variables and non-parametric alternatives (Mann–Whitney U test for two-group comparisons; Kruskal–Wallis for multi-group comparisons) when normality assumptions were violated. Post hoc pairwise comparisons were conducted as appropriate.

**Figure 2:**
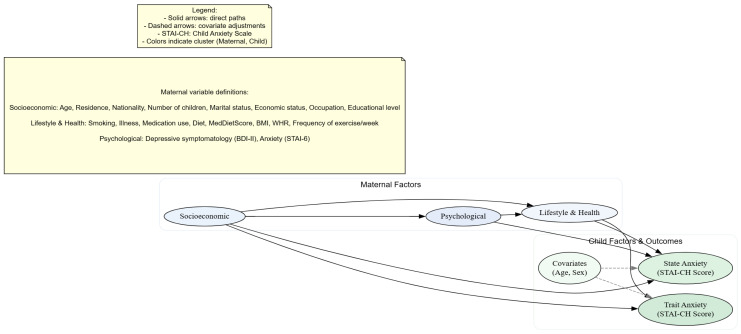
Directed Acyclic Graph (DAG) illustrating hypothesized pathways from maternal characteristics to child trait and state anxiety. Solid arrows indicate direct causal paths between variables, while dashed arrows represent covariate adjustments in the analysis. Colors differentiate variable clusters: maternal factors (blue) and child factors (green).

Candidate covariates for multivariable regression models were pre-specified a priori using a directed acyclic graph (DAG; Figure 2) [56], representing hypothesized causal and confounding pathways. The DAG defined the minimally sufficient adjustment set for all primary analyses. No statistical significance-based variable selection was performed; all DAG-specified covariates were retained to ensure theoretical transparency and reproducibility. *p*-values are reported solely to aid interpretation of effect estimates.

Separate multivariable linear regression models were fitted for child state and trait anxiety. State anxiety reflects maternal proxy-report anxiety observed across all home contexts during the most recent 24-h period (including weekdays and weekends), capturing observable behaviors and emotional states as defined by the STAI-CH. Trait anxiety reflects more stable dispositional tendencies. Continuous covariates were z-standardized (mean = 0, SD = 1), and categorical covariates were dummy-coded. Model outputs include unstandardized coefficients (B), standard errors (SE), standardized coefficients (β), t-values, 95% confidence intervals (CI), *p*-values, sample sizes (n), R^2^, and adjusted R^2^. Degrees of freedom and F-statistics were verified for consistency model specifications (state anxiety: F (8, 191) = 2.397, *p* = 0.017; trait anxiety: F (4, 199) = 8.554, *p* < 0.001).

Given the multistage sampling design, potential clustering of participants was assessed at two hierarchical levels: preschool and geographic region. Intraclass correlation coefficients (ICCs) were estimated using two-level random intercept models, with children (level 1) nested within preschools (level 2). Region-level ICCs were estimated in separate models using geographic region as the grouping factor. Random intercept models were fitted using restricted maximum likelihood (REML). ICCs were effectively zero (<0.001) for all outcomes (state anxiety: school ≈ 0.000002, region ≈ 0.000001; trait anxiety: school ≈ 0.000001, region ≈ 0.000001), supporting the use of standard linear regression without cluster-robust or multilevel corrections.

All analyses were conducted using Statistica software (version 10.0; StatSoft Inc., Tulsa, OK, USA). This analytic framework strengthens methodological transparency, reproducibility, and alignment with contemporary epidemiological standards.

## 3. Results

### 3.1. Descriptive Statistics of the Study Population

The analytical sample consisted of 200 mother-child dyads recruited across multiple regions of Greece. Sociodemographic and household characteristics demonstrated relative homogeneity across key population parameters. The majority of participating mothers were of Greek origin (95.5%), with a smaller proportion representing other nationalities (4.5%). Most participants were actively employed (81.4%), while 18.6% reported current unemployment.

Residential distribution indicated predominance of rural residence (85.5%), with a smaller proportion residing in urban settings (14.5%). Maternal age distribution showed concentration within the 35–42-year category (61%), followed by 23% aged 23–34 years and 16% aged above 43 years. Educational attainment levels were generally high, with 67.5% of mothers reporting tertiary or postgraduate education, 30% secondary education, and 2.5% primary-level education or lower. Marital status distribution indicated that most participants were married (94%), with smaller proportions reporting single (2.5%) or divorced status (3.5%). Family structure was characterized predominantly by smaller household sizes, with 79.5% of mothers reporting one or two children. Families with three to four children accounted for 17% of the sample, while larger families (five or more children) represented 3.5%. Self-perceived household financial status was most frequently reported as moderate (52.5%), followed by low (41%), while only 6.5% reported high economic status.

Regarding child characteristics, females represented 55.5% of the sample, whereas males accounted for 44.5%. Age distribution was concentrated in the 4–5-year group (61.5%), followed by 5–6 years (28.5%) and 6–6.5 years (10%), reflecting the expected demographic structure of preschool-aged populations in Greece.

### 3.2. Anthropometric Profile, Maternal Health Status, and Lifestyle-Related Characteristics of the Study Population

Most mothers participating in the study reported abstinence from tobacco use (79.5%) and absence of clinically diagnosed chronic disease at the time of assessment (80.5%). Despite this generally favorable health status, 16.5% reported ongoing pharmacological treatment, mainly related to chronic disorders such as hypothyroidism, autoimmune thyroid disease (Hashimoto’s thyroiditis), beta-thalassemia carrier status, and autoimmune psoriatic arthritis.

Assessment of maternal body composition showed that over half of the participants were classified within the normal BMI category (57.5%), whereas 27.5% were categorized as overweight and 15.0% as obese. Pronounced shifts in body weight distribution were documented across the gestational period. Before pregnancy, 79.0% of mothers fell within the normal BMI classification, whereas at delivery this proportion decreased markedly to 29.0%. In parallel, the proportion of overweight and obesity increased to 44.5% and 26.5%, respectively.

Evaluation of central and peripheral body measurements demonstrated that 42.5% of mothers presented waist circumference values between 76 and 90 cm, while hip circumference ranged from 96 to 110 cm in 55.5% of participants. WHR values were predominantly within the expected physiological range for adult females (0.76–0.85) in 55.5% of the sample. Height distribution appeared relatively homogeneous, with 55.5% of participants classified within the predefined average height category of the study population.

Regarding child health status, the vast majority of preschool-aged children had no documented medical diagnoses (95.5%) and were not receiving routine medication therapy (98.5%). A small subgroup (4.5%) presented diagnosed conditions. These included neurodevelopmental or developmental disorders (2.0%), such as autism spectrum disorder, attention-deficit/hyperactivity disorder, and language development delay, as well as somatic conditions (2.5%), including allergic disease, asthma, eczema, and iron-deficiency anemia.

### 3.3. Maternal Lifestyle Behaviors and Psychological Status

Maternal health-related behaviors and psychosocial factors were examined as critical exposures within the study’s intergenerational framework. Concerning dietary patterns, only a minority of mothers (4.5%) reported following a specific diet, whereas the vast majority did not adhere to any structured dietary regimen. Evaluation via the MedDietScore indicated that 73.0% of participants demonstrated strong alignment with the Mediterranean dietary pattern, reflecting generally favorable nutritional practices.

Levels of physical activity varied across the cohort. A substantial proportion of mothers (37.5%) reported no engagement in structured exercise, 33.5% participated only sporadically, and 29.0% maintained consistent physical activity routines.

Psychological well-being was assessed through validated instruments capturing depressive symptoms and anxiety. Most mothers (78.0%) exhibited minimal or no depressive symptomatology, whereas 22.0% presented with elevated depressive symptoms. Anxiety was distributed approximately evenly, with 50.5% of participants classified as experiencing elevated anxiety, while 49.5% fell within normative ranges.

Collectively, these findings reveal heterogeneity in maternal lifestyle and mental health profiles. High adherence to the MD coexisted with relatively low engagement in physical activity, alongside a notable subset of mothers exhibiting increased psychological distress, highlighting potential areas for targeted intervention and health promotion.

### 3.4. Children’s State and Trait Anxiety

Emotional functioning in early childhood was evaluated through separate assessment of situational and dispositional anxiety dimensions. Distributional analysis demonstrated that most children were clustered within the low-to-intermediate anxiety spectrum across both constructs.

For state anxiety, which captures transient stress responses to environmental stimuli, the largest proportion of children fell within the moderate range (62%), followed by those presenting low levels (34.5%), while only a small fraction exhibited elevated state anxiety (3%). Regarding trait anxiety, representing relatively stable emotional reactivity patterns, over two-thirds of the sample (68.5%) demonstrated low levels, whereas 26.5% were categorized within the moderate range and a minority (5%) presented elevated trait anxiety.

Taken together, these findings suggest that, within this preschool cohort, clinically concerning anxiety manifestations were limited, with most children demonstrating emotional responses consistent with typical developmental expectations.

### 3.5. Comparative Analysis of Maternal Socio-Demographic, Anthropometric, Psychological, and Lifestyle Characteristics in Relation to Children’s Anxiety Levels

#### 3.5.1. Association Between Maternal Parameters and Children’s State Anxiety

Unadjusted analyses were conducted to examine associations between maternal characteristics and preschool children’s state anxiety levels (Table 1). Normality of distributions were assessed using the Kolmogorov–Smirnov test, and between-group comparisons were performed using the Mann–Whitney U test, as appropriate.

Overall, most maternal demographic, socioeconomic, anthropometric, and lifestyle characteristics were not statistically associated with children’s state anxiety. Maternal age did not demonstrate a statistically significant association with state anxiety (*p* = 0.438); descriptively, lower state anxiety levels were observed among children of mothers aged over 43 years. Place of residence (urban vs. rural; *p* = 0.953) and maternal nationality (*p* = 0.540) were also not statistically associated with children’s state anxiety, although descriptively lower anxiety levels were observed among children of foreign-born mothers.

Family structure, marital status, and household economic status showed no statistically significant associations with children’s state anxiety. Maternal educational attainment was likewise not statistically associated with state anxiety (*p* = 0.854).

A statistically significant association was observed for the number of children in the household (*p* = 0.029). Higher state anxiety levels were observed among children from households with five or more children, whereas lower levels were observed among children from households with three to four children.

Regarding maternal anthropometric measures, no statistically significant associations were identified for weight, height, or BMI. Waist-to-hip ratio (WHR) was significantly associated with children’s state anxiety in unadjusted analyses (*p* = 0.031), with descriptively lower state anxiety levels observed among children of mothers with lower WHR values. This association did not persist in multivariable models.

Maternal lifestyle characteristics, including physical activity frequency, smoking status, illness status, medication use, and dietary adherence assessed using the MedDietScore, were not statistically associated with children’s state anxiety. Although descriptively lower anxiety levels were observed among children of mothers who reported smoking, this association did not reach statistical significance (*p* = 0.730).

In contrast, maternal depressive symptomatology, as assessed by the Beck Depression Inventory, was strongly and statistically associated with children’s state anxiety (*p* < 0.001), with higher state anxiety levels observed among children of mothers reporting elevated depressive symptoms.

The Mann–Whitney U test further demonstrated a statistically significant difference in state anxiety distributions between groups (U = 3322, *p* = 0.013), with children classified as having normal state anxiety exhibiting higher mean ranks (Mean Rank = 106.39) compared to children classified as having elevated state anxiety (Mean Rank = 84.73).

#### 3.5.2. Association Between Maternal Factors and Children’s Trait Anxiety

Unadjusted analyses were conducted to examine associations between maternal characteristics and preschool children’s trait anxiety. Children were categorized into normal and elevated trait anxiety groups (Table 2). Data normality was assessed using the Kolmogorov–Smirnov test, and between-group comparisons were performed using the Mann–Whitney U test.

Overall, the majority of maternal demographic, socioeconomic, anthropometric, and lifestyle characteristics were not statistically associated with children’s trait anxiety. Maternal age (*p* = 0.945), place of residence (*p* = 0.127), nationality (*p* = 0.153), and number of children in the household (*p* = 0.249) showed no statistically significant associations with trait anxiety classification. Although not statistically significant, descriptive patterns indicated a higher proportion of elevated trait anxiety among children of younger mothers, those from larger households, and those whose mothers resided in rural areas. Maternal educational level was also not significantly associated with trait anxiety (*p* = 0.211); descriptively, a higher proportion of children of highly educated mothers were classified within the normal anxiety group (63%) compared with children of less educated mothers (80%).

Maternal anthropometric measures, including weight, height, BMI, and WHR, were not statistically associated with children’s trait anxiety. WHR approached statistical significance but did not meet the conventional threshold (*p* = 0.087); descriptively lower WHR values were observed among mothers of children classified in the normal anxiety group.

Among maternal lifestyle characteristics, smoking status was statistically associated with children’s trait anxiety classification (*p* = 0.005), with a higher proportion of children classified as having normal trait anxiety among mothers who reported smoking.

Maternal psychological characteristics, including depressive symptoms (*p* = 0.206) and anxiety levels (STAI-6, *p* = 0.655) were not statistically associated with children’s trait anxiety. Additional maternal variables, including illness status, medication use, special dietary practices, family economic status, and professional consultation, also showed no statistically significant associations with trait anxiety.

The Mann–Whitney U test demonstrated a statistically significant difference between the two groups (U = 3606.5, *p* = 0.048), with children classified as having normal trait anxiety exhibiting higher mean ranks (Mean Rank = 105.18) compared to children classified as having elevated trait anxiety (Mean Rank = 88.14).

### 3.6. Multivariable Analyses

Two separate multivariable linear regression models were constructed to examine independent associations between maternal characteristics and preschool children’s anxiety, distinguishing between transient (state) and dispositional (trait) anxiety dimensions. This analytic approach allowed for the assessment of maternal sociodemographic, psychological, and anthropometric factors in relation to distinct anxiety profiles in early childhood, while accounting for potential confounding.

#### 3.6.1. Multivariable Linear Regression Analysis of Maternal Characteristics Associated with Children’s State Anxiety

A multivariable linear regression analysis was conducted to examine independent associations between maternal characteristics and preschool children’s state anxiety. The overall model was statistically significant and accounted for 9.1% of the variance in state anxiety (R^2^ = 0.091, adjusted R^2^ = 0.053), F (8, 191) = 2.397, *p* = 0.017 (Table 3), indicating a modest but statistically meaningful explanatory capacity.

Within the model, maternal anxiety was independently and positively associated with children’s state anxiety (B = 1.508, SE = 0.566, β = 0.196, t = 2.666, *p* = 0.008; 95% CI [0.43, 2.66]), such that higher maternal anxiety scores were associated with higher levels of transient anxiety in children. Maternal educational attainment was also independently associated with children’s state anxiety (B = 1.061, SE = 0.509, β = 0.145, t = 2.086, *p* = 0.038; 95% CI [0.08, 2.09]), with higher educational levels corresponding to higher state anxiety scores.

In contrast, maternal age (B = −0.025, SE = 0.060, β = −.029, t = −0.415, *p* = 0.678), maternal WHR (B = −0.001, SE = 0.005, β = −.011, t = −0.155, *p* = 0.877), and maternal depressive symptomatology (B = 0.521, SE = 0.675, β = 0.056, t = 0.771, *p* = 0.441) were not independently associated with children’s state anxiety. Similarly, family size, household economic status, and family structure showed no statistically significant associations with state anxiety, as all corresponding 95% confidence intervals included the null value.

Overall, although the proportion of variance explained by the model was limited, maternal anxiety and maternal educational attainment remained independently associated with preschool children’s state anxiety, whereas maternal age, anthropometric indices, depressive symptoms, family size, economic status, and family structure were not associated with this outcome with the multivariable framework.

#### 3.6.2. Multivariable Linear Regression Analysis of Maternal Characteristics Associated with Children’s Trait Anxiety

A separate multivariable linear regression model was constructed to examine independent associations between maternal characteristics and preschool children’s trait anxiety. The model was statistically significant and accounted for 14.9% of the variance in trait anxiety (R^2^ = 0.149, adjusted R^2^ = 0.132), F (4, 199) = 8.554, *p* < 0.001 (Table 4), indicating moderate explanatory capacity.

Within the model, maternal depressive symptomatology was independently and strongly associated with children’s trait anxiety (B = 3.578, SE = 0.918, β = 0.276, t = 3.897, *p* < 0.001; 95% CI [1.77, 5.39]). Maternal anxiety was also independently associated with trait anxiety (B = 2.088, SE = 0.744, β = 0.194, t = 2.807, *p* = 0.006; 95% CI [0.62, 3.56]), with higher maternal symptom levels corresponding to higher trait anxiety scores in children.

In contrast, maternal smoking status (B = 0.749, SE = 0.887, β = 0.056, t = 0.845, *p* = 0.399; 95% CI [−1.00, 2.50]) and maternal WHR (B = 0.029, SE = 0.553, β = 0.004, t = 0.053, *p* = 0.958; 95% CI [−1.06, 1.12]) were not independently associated with children’s trait anxiety.

Overall, maternal depressive symptoms and maternal anxiety remained independently associated with preschool children’s trait anxiety in the multivariable analysis, whereas matenal smoking behavior and anthropometric indices were not associated with this outcome.

## 4. Discussion

Anxiety represents one of the most frequently observed psychological phenomena in early childhood, with accumulating evidence indicating that clinically relevant symptoms may already be evident during the preschool years. While moderate anxiety is widely regarded as a normative and adaptive emotional response that supports learning and environmental adjustment, persistently elevated anxiety—whether situational or dispositional—has been consistently associated with emotional dysregulation, difficulties in social functioning, and increased vulnerability to later psychopathology [57]. Systematic reviews estimate that approximately 17.6% of preschool-aged children present with psychological difficulties, a substantial proportion of which persist over time, underscoring the importance of early identification of factors associated with anxiety risk [58].

Within this framework, the present study adopted a multifactorial analytical approach to examine associations between maternal characteristics and preschool children’s anxiety, explicitly distinguishing between state (transient, context-dependent) and trait (stable, temperamental) anxiety. This conceptual distinction constitutes a meaningful methodological contribution, as anxiety in early childhood has frequently been examined as a unitary construct or subsumed within broader internalizing symptom dimensions, potentially obscuring differential correlates. To our knowledge, few studies have examined maternal characteristics in relation to state and trait anxiety separately in preschool-aged children, and the present finding adds novel evidence to the international literature.

Multivariable analyses revealed differentiated patterns of association across anxiety dimensions. With respect to trait anxiety, maternal depressive symptomatology demonstrated the strongest independent association, followed by maternal anxiety. This pattern is consistent with extensive literature demonstrating robust associations between maternal depression and children’s internalizing symptoms, including anxiety and broader emotional difficulties [59]. Chronic exposure to maternal depressive symptoms has been associated with alterations in emotional climate and parent-child interactions, which may be linked to the consolidation of anxiety as a relatively stable dispositional characteristics rather than a transient emotional state [57,60]. These findings suggest that interventions targeting maternal depressive symptoms may be particularly relevant for children’s trait anxiety, whereas approaches addressing maternal anxiety may more effectively mitigate situational or context-dependent state anxiety. The independent association between maternal anxiety and child trait anxiety further aligns with theoretical models of intergenerational transmission of anxiety-related vulnerability, emphasizing the role of modeling, shared cognitive-affective processes, and daily interaction patterns [61,62].

In contrast, the multivariable model examining state anxiety yielded a partially distinct profile of associated maternal characteristics. Maternal anxiety remained independently associated with state anxiety, consistent with its relevance to situational emotional reactivity. Higher maternal educational attainment showed a weak positive association with children’s state anxiety. This association should be interpreted cautiously, as it may reflect increased detection or reporting of anxiety symptoms by more-educated mothers or other unmeasured socio-environmental factors. Mechanistic explanations, such as higher parental expectations or sensitivity to performance-related behaviors, remain speculative and warrant further investigation [26,63].

Unadjusted analyses additionally indicated lower state anxiety levels among children of foreign-born mothers. While the association did not persist in adjusted models, it is noteworthy in light of the “immigrant paradox”, whereby children of first-generation immigrant parents have been observed to exhibit more favorable mental health profiles in certain contexts [64,65]. Although evidence in preschool-aged populations remains limited, this observation is consistent with international findings and highlights the potential relevance of sociocultural and contextual factors that warrant further investigation.

Several maternal characteristics, including family structure, number of children, maternal age, socioeconomic status, geographic residence, anthropometric indices, smoking behavior, and dietary adherence, were associated with children’s anxiety in unadjusted analyses but did not retain statistical significance after multivariable adjustments. This attenuation suggests that these factors may be indirectly related to child anxiety or confounded by maternal psychological characteristics. For instance, associations between child anxiety and family structure or socioeconomic disadvantage may reflect increased maternal stress or emotional burden rather than independent associations with child anxiety [66,67,68,69]. Similarly, mixed findings in the literature regarding family size—ranging from potentially protective effects in supportive contexts [70] to elevated stress in socioeconomically constrained environment [71,72], underscore the importance of contextual moderators that may not independently account for anxiety outcomes once proximal psychological variables are considered.

Maternal anthropometric measures, including WHR, and lifestyle behaviors such as smoking were associated with child anxiety in preliminary analyses but were not independently associated in adjusted models. Although emerging research suggests potential links between maternal physical health indicators and offspring emotional outcomes [73,74], the present findings indicate that these associations do not persist after accounting for maternal psychological well-being.

Taken together, the findings indicate that maternal psychological characteristics, particularly depressive symptoms and anxiety, are most consistently associated with preschool children’s anxiety, with depressive symptoms more strongly related to trait anxiety and maternal anxiety associated with both state and trait dimensions. In contrast, sociodemographic, socioeconomic, and anthropometric characteristics appear to play secondary or indirect roles that diminish after adjustment for maternal psychological factors. By differentiating between state and trait anxiety, the present study provides refined insight into early anxiety profiles and highlights the importance of tailoring prevention and intervention efforts to specific anxiety dimensions. Approaches addressing maternal depressive symptoms may be particularly relevant for anxiety dispositions, whereas strategies focusing on maternal anxiety may be more closely aligned with children’s situational anxiety responses.

The present study demonstrates several methodological strengths that enhance the credibility, rigor and interpretability of its findings. First, the sampling strategy drew from geographically diverse regions across Greece, including both urban and rural areas. This approach increases the external validity of the results and supports broader national representativeness, an important consideration given the regional variability often observed in early childhood health and psychosocial factors. Second, the study employed a multifactorial analytical framework incorporating sociodemographic, anthropometric, psychosocial, and lifestyle variables. By examining multiple domains within a single model, the analysis reduced the risk of omitted-variable bias and allowed for a more nuanced understanding of the correlates of early childhood anxiety. Such an integrative approach aligns with contemporary developmental psychopathology frameworks emphasizing the interplay of biological, contextual, and familial factors. Third, the application of validated psychometric instruments, administered following standardized procedures, strengthened construct validity and enhanced confidence in the measurement of maternal and child psychological characteristics. The incorporation of standardized anthropometric assessments performed by trained personnel further minimized measurement error and increased the reliability of the physical health data. Additionally, the differentiation between state and trait anxiety in preschool-aged children represents a notable contribution. Few studies in this age range adopt this distinction due to developmental and methodological complexities, and the present findings offer preliminary but meaningful insight into early emotional profiles. The inclusion of both anxiety dimensions provides a richer understanding of how maternal factors relate differentially to transient versus more stable aspects of child emotional functioning.

Nevertheless, several limitations should be acknowledged. First, the cross-sectional design precludes causal inference and limits conclusions regarding temporal ordering of the observed associations. A further methodological limitation concerns the single-informant design, as mothers reported both their own psychological characteristics and their preschool children’s anxiety. This shared-method approach introduces a potential internal validity threat, given that maternal psychological distress may influence perception and reporting of child behavior, thereby inflating associations between maternal and child variables.

In addition, although the STAI-CH was administered following established procedures for proxy-report in preschool populations, the use of maternal reports to assess both state and trait anxiety raises conceptual challenges. Mothers were instructed to evaluate state anxiety based on how their child appeared to feel during the assessment period and in the immediate present, consistent with standard STAI-State guidelines. However, transient emotional states are less directly accessible to proxy observers, and parents may rely on their child’s general behavioral tendencies rather than momentary fluctuations. Consequently, proxy-report may partially blur the distinction between situational (state) and dispositional (trait) anxiety, potentially reducing the precision with which “state anxiety” is operationalized in this age group. This limitation should be considered when interpreting the findings, particularly the associations between maternal depressive or anxiety symptoms and child anxiety levels. Future studies employing multi-informant designs (e.g., teacher ratings, observational protocols, physiological indices) are needed to disentangle genuine child anxiety presentations from potential informant-related biases.

Additional limitations should also be noted. Reliance on self-report measures, which may introduce recall or social desirability effects, and the exclusive focus on maternal characteristics restricts the ability to account for the broader caregiving environment, including parental, family-system, and school-related influences. The sample size was modest (n = 200), and regional recruitment resulted in uneven representation across Greek regions, potentially limiting generalizability. Moreover, personal acquaintance of researchers with some participants may have inadvertently contributed to socially desirable responding. Finally, specific cultural and societal factors in Greece—such as parenting norms linked to maternal educational attainment, expectations regarding emotional expression, and family dynamics—may have shaped the observed associations. The contextual influences warrant cautious interpretation and highlight the importance of longitudinal, multi-informant research to clarify temporal pathways and inform targeted interventions supporting maternal mental health and early childhood anxiety.

## 5. Conclusions

This study provides novel evidence on the associations between maternal characteristics and preschool children’s anxiety, explicitly distinguishing between trait and state dimensions. Maternal depressive symptomatology was independently associated with higher levels of trait anxiety, whereas maternal anxiety was associated with both trait and state anxiety, highlighting differential patterns across anxiety dimensions.

Other maternal characteristics, including anthropometric, socio-demographic, and family-related factors such as maternal age, educational attainment, family structure, number of children in the household, geographic residence, WHR, and foreign-born status, were related to child anxiety in unadjusted analyses; however, these associations did not consistently retain statistical significance in multivariable models. This pattern suggests that such factors may influence child anxiety indirectly or in context-dependent ways, primarily through proximal maternal psychological characteristics

The observed association between foreign-born maternal status and lower child state anxiety aligns with prior evidence indicating more favorable mental health outcomes among children of first-generation immigrant mothers, although this relationship did not persist after adjustment. Collectively, the findings underscore the primary role of maternal psychological characteristics in early childhood anxiety, while broader sociodemographic and anthropometric factors appear to exert secondary or indirect effects. By differentiating between state and trait anxiety, this study contributes a more nuanced understanding of early anxiety profiles and emphasizes the importance of considering distinct anxiety dimensions in research and preventive interventions.

It is worth noted that these findings should be interpreted with caution. The proportion of variance explained by the models was modest (R^2^ < 0.15), indicating that the majority of variability in child anxiety does not seem to be fully explained by the measured maternal factors. Furthermore, exclusive reliance on maternal proxy-reports introduces the potential for single-informant bias, which may reduce the precision of measuring both state and trait anxiety. Consequently, the reported associations should be regarded as correlational rather than causal. Future longitudinal studies incorporating multi-informant assessments and examining potential mediating mechanisms are warranted to clarify temporal dynamics and to inform evidence-based strategies supporting maternal psychological well-being and early childhood emotional development within a multilevel framework.

## Figures and Tables

**Table 1:** Statistical correlation between maternal characteristics and children’s state anxiety.

Independent Maternal Variables		n	Normal State Anxiety Level	Elevated State Anxiety Level	*p*
Age	23–34 years old	46	31 (67.4%)	15 (32.6%)	0.438
	35–42 years old	122	81 (66.4%)	41 (33.6%)	
	43+ years old	32	25 (78.1%)	7 (21.9%)	
Permanent residence	Urban	29	20 (69%)	9 (31%)	0.953
	Rural	171	117 (68.4%)	54 (31.6%)	
Nationality	Greek	191	130 (68.1%)	61 (31.9%)	0.540
	Non-Greek	9	7 (77.8%)	2 (22.2%)	
Number of children	1–2 children	159	108 (67.9%)	51 (32.1%)	0.029
	3–4 children	34	27 (79.4%)	7 (20.6%)	
	5+ children	7	2 (28.6%)	5 (71.4%)	
Weight	44–60 kg	72	52 (72.2%)	20 (27.8%)	0.441
	61–75 kg	76	48 (63.2%)	28 (36.8%)	
	75+ kg	52	37 (71.2%)	15 (28.8%)	
Height	1.50–1.60 m	60	39 (65%)	21 (35%)	0.777
	1.61–1.70 m	111	78 (70.3%)	33 (29.7%)	
	1.71+ m	29	20 (69%)	9 (31%)	
BMI	Normal weight	115	78 (67.8%)	37 (32.2%)	0.896
	Overweight	55	39 (70.9%)	16 (29.1%)	
	Obesity	30	20 (66.7%)	10 (33.3%)	
Waist circumference	60–75 cm	57	40 (70.2%)	17 (29.8%)	0.942
	76–90 cm	85	58 (68.2%)	27 (31.8%)	
	91+ cm	58	39 (67.2%)	19 (32.8%)	
Hip circumference	70–95 cm	56	38 (67.9%)	18 (32.1%)	0.766
	96–110 cm	111	78 (70.3%)	33 (29.7%)	
	111+ cm	33	21 (63.6%)	12 (36.4%)	
WHR	≥0.75	35	30 (85.7%)	5 (14.3%)	0.031
	0.76–0.85	111	69 (62.2%)	42 (37.8%)	
	0.86+	54	38 (70.4%)	16 (29.6%)	
Pre-pregnancy weight	44–55 kg	60	43 (71.7%)	17 (28.3%)	0.613
	56–70 kg	104	68 (65.4%)	36 (34.6%)	
	71+ kg	36	26 (72.2%)	10 (27.8%)	
Weight before delivery	50–65 kg	47	30 (63.8%)	17 (36.2%)	0.545
	66–80 kg	96	65 (67.7%)	31 (32.3%)	
	81+ kg	57	42 (73.7%)	15 (26.3%)	
Frequency of exercise/week	None	75	50 (66.7%)	25 (33.3%)	0.792
	A little	67	48 (71.6%)	19 (28.4%)	
	Often	58	39 (67.2%)	19 (32.8%)	
Smoking	Yes	41	29 (70.7%)	12 (29.3%)	0.730
	No	159	108 (67.9%)	51 (32.1%)	
Illness	Yes	39	27 (69.2%)	12 (30.8%)	0.913
	No	161	110 (68.3%)	51 (31.7%)	
Medication use	Yes	33	22 (66.7%)	11 (33.3%)	0.804
	No	167	115 (68.9%)	52 (31.1%)	
Diet (type)	Non-specific diet	191	131 (68.6%)	60 (31.4%)	0.904
	Special diet	9	6 (66.7%)	3 (33.3%)	
Marital status	Single	5	2 (40%)	3 (60%)	0.378
	Married	188	130 (69.1%)	58 (30.9%)	
	Divorced	7	5 (71.4%)	2 (28.6%)	
Economic status	Low	82	52 (63.4%)	30 (36.6%)	0.423
	Medium	105	76 (72.4%)	29 (27.6%)	
	High	13	9 (69.2%)	4 (30.8%)	
Occupation	Unemployed	37	27 (73%)	10 (27%)	0.502
	Employed	162	109 (67.3%)	53 (32.7%)	
Educational level	Low	5	4 (80%)	1 (20%)	0.854
	Medium	60	41 (68.3%)	19 (31.7%)	
	High	135	92 (68.1%)	43 (31.9%)	
MedDietScore	Low adherence	54	37 (68.5%)	17 (31.5%)	0.997
	High adherence	146	100 (68.5%)	46 (31.5%)	
Depressive symptomatology (BDI-II)	Minimal depressive symptoms	156	117 (75%)	39 (25%)	<0.001
	Depressive symptoms	44	20 (45.5%)	24 (54.5%)	
Anxiety (STAI-6)	Normal level	99	78 (69%)	35 (31%)	0.820
	High level	10	34 (70.8%)	14 (29.2%)	
Professional consultation about diet	Yes	25	16 (64%)	9 (36%)	0.605
	No	175	121 (69.1%)	54 (30.9%)	

**Table 2:** Statistical correlation between maternal characteristics and children’s trait anxiety.

Independent Maternal Variables		n	Normal Level of Trait Anxiety	Elevated Trait Anxiety Level	*p*
Age	23–34 years old	46	30 (65.2%)	16 (34.8%)	0.945
	35–42 years old	122	82 (67.2%)	40 (32.8%)	
	43+ years old	32	22 (68.8%)	10 (31.3%)	
Permanent residence	Urban	29	23 (79.3%)	6 (20.7%)	0.127
	Rural	171	111 (64.9%)	60 (35.1%)	
Nationality	Greek	191	126 (66%)	65 (34%)	0.153
	Non-Greek	9	8 (88.9%)	1 (11.1%)	
Number of children	1–2 children	159	111 (69.8%)	48 (30.2%)	0.249
	3–4 children	34	19 (55.9%)	15 (44.1%)	
	5+ children	7	4 (57.1%)	3 (42.9%)	
Weight	44–60 kg	72	46 (63.9%)	26 (36.1%)	0.625
	61–75 kg	76	54 (71.1%)	22 (28.9%)	
	75+ kg	52	34 (65.4%)	18 (34.6%)	
Height	1.50–1.60 m	60	40 (66.7%)	20 (33.3%)	0.971
	1.61–1.70 m	111	74 (66.7%)	37 (33.3%)	
	1.71+ m	29	20 (69%)	9 (31%)	
BMI	Normal weight	115	80 (69.6%)	35 (30.4%)	0.431
	Overweight	55	33 (60%)	22 (40%)	
	Obesity	30	21 (70%)	9 (30%)	
Waist circumference	60–75 cm	57	38 (66.7%)	19 (33.3%)	0.998
	76–90 cm	85	57 (67.1%)	28 (32.9%)	
	91+ cm	58	39 (67.2%)	19 (32.8%)	
Hip circumference	70–95 cm	56	37 (66.1%)	19 (33.9%)	0.934
	96–110 cm	111	74 (66.7%)	37 (33.3%)	
	111+ cm	33	23 (69.7%)	10 (30.3%)	
WHR	≥0.75	35	29 (82.9%)	6 (17.1%)	0.087
	0.76–0.85	111	70 (63.1%)	41 (36.9%)	
	0.86+	54	35 (64.8%)	19 (35.2%)	
Pre-pregnancy weight	44–55 kg	60	38 (63.3%)	22 (36.7%)	0.490
	56–70 kg	104	69 (66.3%)	35 (33.7%)	
	71+ kg	36	27 (75%)	9 (25%)	
Weight before delivery	50–65 kg	47	28 (59.6%)	19 (40.4%)	0.457
	66–80 kg	96	66 (66.8%)	30 (31.3%)	
	81+ kg	57	40 (70.2%)	17 (29.8%)	
Frequency of exercise/week	None	75	46 (61.3%)	29 (38.7%)	0.418
	A little	67	47 (70.1%)	20 (29.9%)	
	Often	58	41 (70.7%)	17 (29.3%)	
Smoking	Yes	41	35 (85.4%)	6 (14.6%)	0.005
	No	159	99 (62.3%)	60 (37.7%)	
Illness	Yes	39	26 (66.7%)	13 (33.3%)	0.961
	No	161	108 (67.1%)	53 (32.9%)	
Medication use	Yes	33	20 (60.6%)	13 (39.4%)	0.393
	No	167	114 (68.3%)	53 (31.7%)	
Diet (type)	Non-specific diet	191	128 (67%)	63 (33%)	0.983
	Special diet	9	6 (66.7%)	3 (33.3%)	
Marital status	Single	5	3 (60%)	2 (40%)	0.537
	Married	188	125 (66.5%)	63 (33.5%)	
	Divorced	7	6 (85.7%)	1 (14.3%)	
Economic status	Low	82	52 (63.4%)	30 (36.6%)	0.314
	Medium	105	71 (67.6%)	34 (32.4%)	
	High	13	11 (84.6%)	2 (15.4%)	
Occupation	Unemployed	37	26 (70.3%)	11 (29.7%)	0.673
	Employed	162	108 (66.7%)	54 (33.3%)	
Educational level	Low	5	4 (80%)	1 (20%)	0.211
	Medium	60	45 (75%)	15 (25%)	
	High	135	85 (63%)	50 (37%)	
MedDietScore	Low adherence	54	39 (72.2%)	15 (27.8%)	0.339
	High adherence	146	95 (65.1%)	51 (34.9%)	
Depressive symptomatology (BDI-II)	Minimal depressive symptoms	156	108 (69.2%)	48 (30.8%)	0.206
	Depressive symptoms	44	26 (59.1%)	18 (40.9%)	
Anxiety (STAI-6)	Normal level	99	76 (67.3%)	37 (32.7%)	0.655
	High level	10	34 (70.8%)	14 (29.2%)	
Professional consultation about diet	Yes	25	18 (72%)	7 (28%)	0.570
	No	175	116 (66.3%)	59 (33.7%)	

**Table 3:** Multivariable linear regression examining associations between maternal characteristics and preschool children’s state anxiety.

Maternal Variables	B	SE	β	t	95% CI	*p*-Value	n
Constant	24.708	3.814	—	6.478	14.56–30.17	<0.001	200
Age	−0.025	0.060	−0.029	−0.415	−0.14–0.09	0.678	200
WHR	−0.001	0.005	−0.011	−0.155	−0.10–0.01	0.877	200
Depressive symptoms (BDI-II)	0.521	0.675	0.056	0.771	−0.79–1.87	0.441	200
Anxiety (STAI-6)	1.508	0.566	0.196	2.666	0.43–2.66	0.008	200
Number of children	0.149	0.291	0.036	0.511	−0.42–0.73	0.610	200
Socioeconomic status	−0.675	0.457	−0.105	−1.476	−1.61–0.20	0.141	200
Marital status	−0.507	1.090	−0.032	−0.465	−0.97–1.60	0.642	200
Educational level	1.061	0.509	0.145	2.086	0.08–2.09	0.038	200

Note. *R*^2^ = 0.091, Adjusted *R*^2^ = 0.053, *F* (8, 191) = 2.397, *p* = 0.017.

**Table 4:** Multivariable linear regression examining associations between maternal characteristics and preschool children’s trait anxiety.

Maternal Variables	B	SE	β	t	95% CI	*p*-Value	n
Constant	19.082	2.393	-	7.974	14.36–23.80	<0.001	200
Smoking	0.749	0.887	0.056	0.845	−1.0–2.50	0.399	200
WHR	0.029	0.553	0.004	0.053	−1.06–1.12	0.958	200
Depressive symptoms (BDI-II)	3.578	0.918	0.276	3.897	1.77–5.39	<0.001	200
Anxiety (STAI-6)	2.088	0.744	0.194	2.807	0.62–3.56	0.006	200

Note. *R*^2^ = 0.149, Adjusted *R*^2^ = 0.132, *F* (4, 199) = 8.554, *p* < 0.001.

## Data Availability

The original contributions presented in this study are included in the article. Further inquiries can be directed to the corresponding author.
